# Supplementary material for: Regulatory Landscape Enrichment Analysis (RLEA) using gaiaAssociation
Source: bioRxiv. 2023 Oct 16:2023.10.11.561933. Preprint. [Version 1] doi: 10.1101/2023.10.11.561933 (PMC10614734; doi:10.1101/2023.10.11.561933)
Supplement: 2 [file NIHPP2023.10.11.561933v1-supplement-2.pdf]

bioRxiv preprint doi: https://doi.org/10.1101/2023.10.11.561933; this version posted October 16, 2023. The copyright holder for this preprint (which was not certified by peer review) is the author/funder, who has granted bioRxiv a license to display the preprint in perpetuity. It is made available under aCC-BY 4.0 International license.

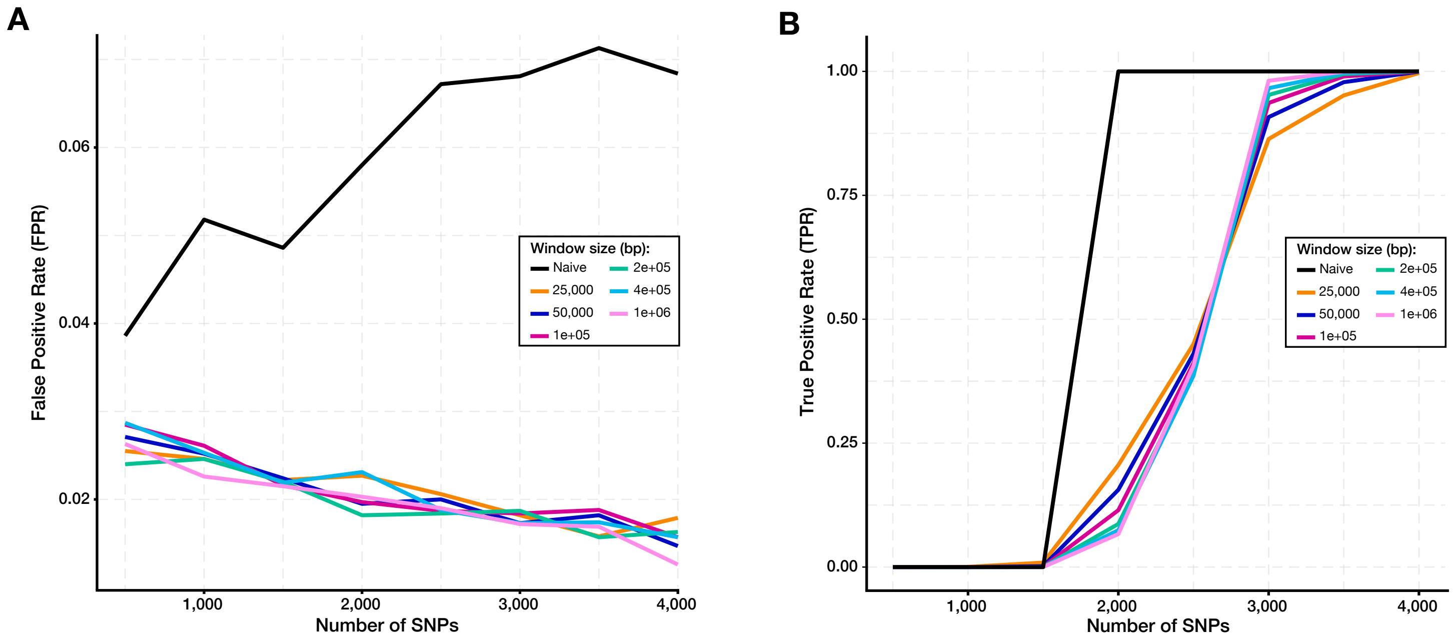

Supplementary Figure 1: 100,000 random simulations of RLEA demonstrate that increased SNPs is inversely related to false positive rate (FPR) and positively corelated to true positive rate (TPR). **(A)** FPR declines as the number of SNPs increases and window size is defined. RLEA FPR was calculated for varying window sizes ranging from 25,000-1,000,000 base pairs (bp) in length. The naïve window size (black line) represents the whole genome in the absence of window selection. **(B)** Increased SNP number and window size are directly correlated with TPR in RLEA. The naïve window TPR plateaus before all defined windows since decreased window sizes are associated with increasingly conservative enrichment. All SNPs in this simulation were selected at random and tested for enrichment in real ATAC-seq peaks.
